# Study on Dual Channel Lateral Field Excitation Quartz Crystal Microbalance for Measuring Liquid Electrical Properties

**DOI:** 10.3390/s19051253

**Published:** 2019-03-12

**Authors:** Jinxing Liang, Debo Kong, Chaolin Liu

**Affiliations:** Key Laboratory of Micro-Inertial Instrument and Advanced Navigation Technology, Ministry of Education, School of Instrument Science and Engineering, Southeast University, Nanjing 210096, China; 103008512@seu.edu.cn (D.K.); 220152742@seu.edu.cn (C.L.)

**Keywords:** dual channel, LFE, QCM, permittivity, conductivity, temperature

## Abstract

Lateral field excitation quartz crystal microbalance (LFE-QCM) can detect both the electrical properties (conductivity and permittivity) and mechanical properties (viscosity and density) of the liquid. In practical applications for detecting electrical properties, the viscosity and density of the liquid will also change. This research proposed a dual-channel LFE-QCM for reducing the influence of density and viscosity. The sensing layer of one resonant element is almost bare, and the other is covered by a metal film as a reference. Different organic solutions and NaCl solution were used to study the influence of mechanical properties and the temperature on electrical properties. The experimental results demonstrate that the dual-channel LFE-QCM is necessary for properly detecting electrical properties of the liquid.

## 1. Introduction

Quartz crystal microbalance (QCM) is a piezoelectric device based on the piezoelectric effect of materials. QCM sensitively measures the mass change on the surface of the electrode. QCM is a well-known high precision resonator with a measurement accuracy in the nano-gram range. The sensor has a high sensitivity, simple structure, and can be used in real time without interruption in gas or liquid environments [1,2,3].

In the thickness shear mode (TSM), AT-cut quartz crystal microbalance sensors with standard electrode geometry have been widely used in liquid phase chemical sensing applications [4]. The detection mechanism is mainly based on mechanical loading effects such as mass, density, and viscosity. According to research by Sauerbrey and Kanazawa, the resonant frequency shifts under the influence of mechanical load, viscosity, and density of a Newtonian liquid, which is expressed as [5,6,7,8]:

(1) $$\Delta f=-\frac{2f_{0}^{2}\Delta m}{A\sqrt{\mu_{q}\rho_{q}}}-f_{0}{}^{\frac{3}{2}}\sqrt{\frac{\rho_{l}\eta_{l}}{\pi \rho_{q}\mu_{q}}}$$

where Δ*f* represents the frequency shift; *f*_0_ is the fundamental resonant frequency; *μ_q_* and *η_q_* represent shear modulus of the AT-cut (2.947 × 10^11^ g∙cm^−1^ s^−2^) and the density of crystal for quartz (2.648 g∙cm^−3^), respectively; Δ*m* is the attached mass; *A* is the piezoelectrically active area; and *ρ_l_* and *η_l_* represent the liquid density and viscosity, respectively.

Until 2000, QCM was usually used as a mass frequency sensor. Since then, studies reported that QCM can directly detect liquid mechanical properties and electrical properties. Hempel et al. [9], Wang et al. [10], and other groups [11,12,13] conducted a series of theoretical analysis and research. A literature review also introduced many practical applications of and prospects for lateral field excitation QCM [14].

**Figure 1:**
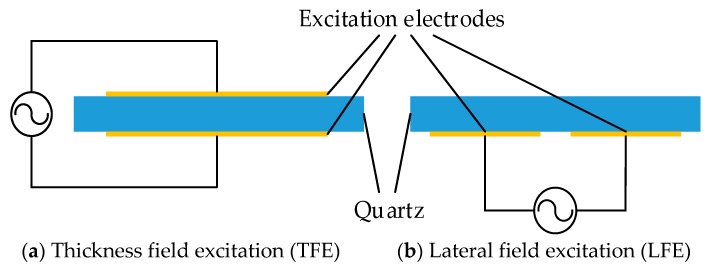
Two kinds of QCM with different structures.

There are two different QCM structures: thickness field excitation QCM (TFE-QCM) and lateral field excitation QCM (LFE-QCM), as shown in the Figure 1. The piezoelectric bulk acoustic wave sensor of the lateral field excitation mode has different electrode structures in addition to the thickness field excitation sensor, and its two electrodes are on the same principal plane of the crystal substrate. This kind of electrode structure enables the electrical field to penetrate into the liquid. Therefore, LFE-QCM has extremely high sensitivity to electrical properties in the liquid, and the mechanical properties similar to the TFE sensor [15,16,17,18]. At present, many studies have been completed on LFE-QCM, which has been shown to have many advantages over traditional QCM in practical applications. The study of the lateral field excitation electrode gap by Hu et al. proved that the wider the gap, the lower the sensitivity to the solution properties, and has the greater electric field penetration into the liquid when the sensing layer is completely exposed [19]. Abe et al. confirmed this finding. A lateral field excitation QCM with a metal film on the sensing layer was designed [20]. This QCM could not detect the electrical properties of the solution when compared with the ordinary lateral field excitation QCM.

The temperature effect cannot be ignored during measurement of a liquid [21]. Liquid mechanical properties such as density, viscosity, and conductivity, are greatly affected by the temperature of the liquid. When used in a liquid, the QCM resonator’s quality factor (Q value) drops sharply due to damping. Liquid viscosity reduces frequency stability. Small mechanical properties and environmental fluctuations result in large frequency drifts, and thereby inaccurate measurements. In theory, the impact of environmental factors can be eliminated by arranging another QCM as a reference [22]. Both Winters et al. [23] and Abe et al. [24] designed two or more independent QCMs on the same quartz blank, etching a quartz groove between two QCM bays. In another study the QCM was individually designed as an inverted step structure to isolate coupling [25]. However, the two channels were not provided for reference.

Although the above structure has been proven to have a good effect, in practical applications, the influence of the liquid electrical properties is generally greater than the influence of the liquid mechanical properties to the resonant frequency, so the influence of mechanical properties is often neglected in the research on detecting electrical properties. To reduce the influence of mechanical properties to the resonant frequency and separately study the influence of the electrical properties of the solution on the resonator, we designed a dual-channel LFE-QCM. One QCM, as a reference, was set to measure only the mechanical properties of the liquid and the other QCM, as a test, was set to measure all the properties from the liquid. When detecting liquids, factors such as ambient temperature can easily affect the nature of the liquid itself, thereby affecting the resonant frequency of the QCM. Therefore, the dual-channel resonant elements on the same wafer can reduce these effects. 

Metamorphism of oil in industrial production and daily life affects our lives, such as through transportation, especially as the moisture content of oil likely causes metamorphism of oil. Therefore, the oil needs to be replaced or supplemented regularly. Oil detection technology can be used to evaluate the state of the oil quality and judge whether it needs to be replaced, but few sensors are sufficiently small and can monitor the oil in real time, which makes the current sensors’ use inconvenient. The dual-channel LFE-QCM designed in this paper not only meets the requirements of miniaturization and real-time monitoring, but also eliminates the interference of environmental temperature and other factors, providing a good method for oil detection. Based on the high sensitivity of LFE-QCM to the electrical properties of the liquid, the water quality of groundwater or rivers and lakes can be judged using this device via the all-weather monitoring of the liquid conductivity. The design of the reference resonator can eliminate the influence of different temperatures in one day.

In the experiment, the organic solution was selected as the test solution because its relative permittivity is different, and its viscosity and density are not directly related to the relative permittivity, and they are widely used in the research with lateral field excitation QCM on electrical property as the detection solution. Either NaCl or KCl are the commonly used solution for studying electrical properties about conductivity, so NaCl was selected as the temperature test solution [18,26]. 

## 2. Experimental Materials and Methods

### 2.1. Design and Fabrication

The two resonant elements of the dual channel LFE-QCM are, respectively, QCM-T (test) resonant element and QCM-R (reference) resonant elements. Both resonant elements adopt the lateral field excitation mode, and the metal excitation electrodes are all on the same side. There are many kinds of lateral field excitation electrode geometry, such as the semicircular, half-moon, and T structures. Different electrode structures have different sensitivities to the frequency response. According to the research, the excitation electrode of the half-moon structure is the most sensitive and excited electrode for detection. The best sensitivity occurs when the electrode gap is parallel to the x-axis of the quartz crystal [20,27,28]. Therefore, we chose the half-moon structure and the gap was designed parallel to the x-axis.

**Figure 2:**
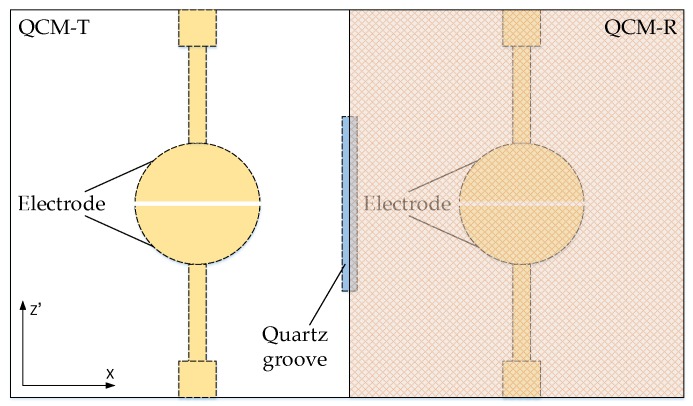
Schematic structure of the dual-channel LFE-QCM.

The QCM-R resonant element covered the quartz blank with an all-metal film to form an electric field shield such that the electric field did not penetrate into the liquid medium [20]. Therefore, the frequency shift caused by the QCM-R resonator was only affected by the viscosity and density of solution. The lateral electric field can easily penetrate into the liquid medium because the upper surface of the QCM-T resonator was approximately bare [19]. The frequency shift caused by the QCM-T resonator not only contained the information about the viscosity and density of the solution, but also the information about the permittivity and conductivity. The influence of the liquid electrical properties on the QCM in the experiment was due to the difference in the frequency between the two resonant elements. Figure 2 depicts the schematic structure of the dual-channel LFE-QCM. The dotted parts show the bottom of the quartz blank. The orange grid area was the metal film on the surface of the quartz blank, which was the QCM-R. The quartz crystal had a single-sided, concave structure, where a quartz groove was etched in a non-oscillating region between the two excitation electrodes to suppress frequency interference.

**Figure 3:**
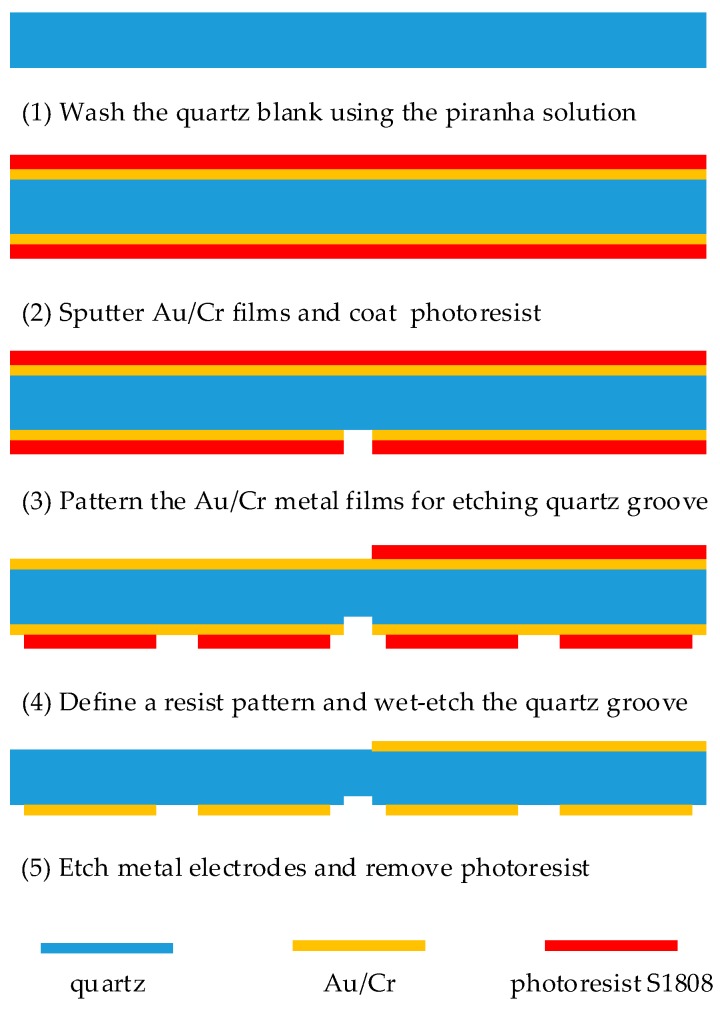
Quartz wet-etching process flow diagram.

The separate AT-cut quartz crystal chip used in this study was designed in a rectangular shape with dimensions of 13.5 mm × 8 mm. The starting blank was 40 mm × 40 mm, and 100 μm thick with double polished planar faces. The fundamental frequency of the QCM resonator was about 16 MHz. The metal excitation electrode had a half-moon structure with a radius of 1.25 mm. Both the metal excitation electrode and the metal film were composed of an Au/Cr double-layer metal film, and gold film was used as a main electrode and a thin chromium film as an adhesion layer between the quartz and the gold film. The quartz groove was 3 mm × 50 μm and 20 μm deep. Five single dual-channel QCM chips could be cut out from one quartz blank. All quartz blanks were processed with the quartz wet etching process, and Figure 3 depicts the process diagram. (1) The quartz blank was washed using piranha solution (H_2_SO_4_:H_2_O_2_ = 3:1); (2) Au/Cr bi-layer films were sputtered on both sides of the quartz blank, followed by the photoresist coating; (3) the Au/Cr bi-layer films on the area of quartz groove were patterned for etching the quartz groove; (4) a resist pattern was formed on both sides of the blank, followed by wet-etching the quartz groove using a saturated ammonium bifluoride solution at 85 °C; (5) then, the Au/Cr bi-layer films were etched to form electrodes and the photoresist was removed, followed by dicing the quartz blank.

### 2.2. Evaluation

**Figure 4:**
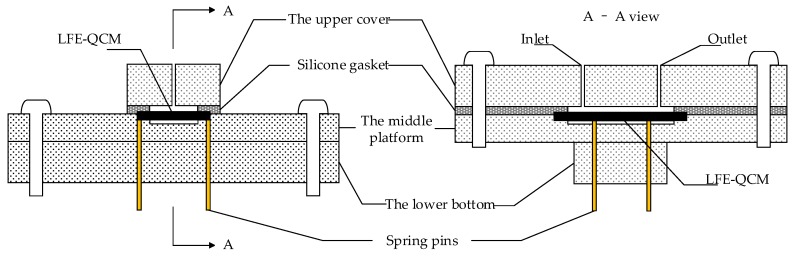
The flow cell structure diagram.

Flow cells were fabricated from polydimethylsiloxane (PDMS) or metal material referencing from Michalzik et al. [29,30] and Sagmeister et al. [31]. We chose to use polymethyl methacrylate (PMMA) to produce the flow cell, referring to the work from Liang et al. [32], because PMMA has better light transmission than metal for observing the flow of liquid, and the produced flow cell can be reused many times. The structure of the flow cell was divided into three parts: the upper cover, the middle platform, and the lower bottom, as shown in Figure 4.

During installation, a quartz blank was first placed in the rectangular recess in the center of the middle platform, and both long sides of the rectangular recess had two electrode access apertures. The side of the quartz blank with the excitation electrode was close to the middle platform, and the other side with the floating metal electrode was connected upward to the upper cover and the silicone gasket. The depth of the rectangular recess ensured the degree of the tightness and reduced the high mechanical strength of the PMMA material itself to the quartz blank. The upper cover had a sample inlet and an outlet with plastic hollow hoses to facilitate the connection of the syringe to allow the solution to circulate. Four small holes corresponded to the electrode access apertures in the lower bottom to lead out the electrodes. The electrodes were extracted using contact spring pins to avoid damaging to the quartz blank or electrode using conventional welding methods, which was beneficial to the reusability of the flow cell.

An impedance analyzer 4294A (Agilent Technologies Inc., Santa Clara, CA, USA) was used to measure the vibration properties including resonance frequency, Q value, conductance, and equivalent circuit parameters. A high frequency dual-channel oscillator system was developed as introduced before [22]. The flow injection system consisted mainly of a syringe pump, a loop, an injector, a flow cell, and a measuring instrument. The measuring instrument can be composed of an impedance analyzer alone or the oscillating circuit with an external frequency counter.

## 3. Results and Discussion

**Figure 5:**
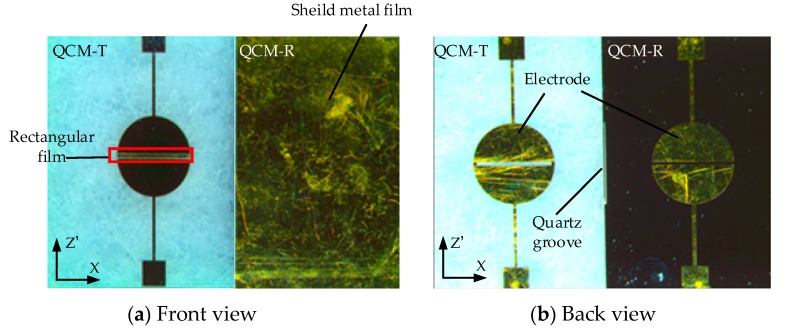
Example pictures of the fabricated dual-channel LFE-QCM chip.

Figure 5 depicts the dual-channel lateral field excitation QCM. On one side of the sensing layer, the QCM-R was shielded by a metal film. The sensing layer, also composed of Au/Cr films, of the QCM-T added a small rectangular film to stimulate the energy trapping and improve the stability of resonance (Figure 5a) [33,34]. Figure 5b shows the back electrode, which was composed of four half-moon electrodes.

The excitation electrode was first connected to the impedance analyzer through spring pins to measure the corresponding parameters of each resonant element of the dual-channel LFE-QCM, ensuring that the two resonant elements could vibrate independently and oscillate in isolation. The energy loss of mechanical vibration of the QCM in the liquid phase was huge, and the vibration attenuation was serious. Therefore, whether stable vibration could be maintained in the liquid phase is crucial for later research with other solutions.

The two resonant elements of LFE-QCM could oscillate independently, indicating that the design of the chip structure and the selection of the flow cell were successful and could be used for subsequent test work. The quality factor (Q value) in the air was 30,000 or more, as shown in Table 1, which proved that the resonant element could generate stable oscillation in air. The Q value of the QCM-R resonant element was higher than that of QCM-T because the QCM-R resonant element’s sensing layer was completely covered by the metal film and the shear wave vibration was concentrated in the quartz blank, resulting in a larger energy-trapping effect. The QCM-R resonant element covering the metal film comprehensively enhanced the vertical component in the lateral electric field, resulting in an order of magnitude difference in conductance values. The Q value of LFE-QCM under deionized water was more than 1100. According to experience, the Q value of QCM generally needs to be greater than 1000 when used in a liquid phase. Therefore, the above experiments verified that the chip used in this experiment can be used for detection in liquid environments. Comparing the conductance values of the two resonant elements in the air, the conductance of the QCM-R was 200 times higher than that of the QCM-T. In water, the conductance of QCM-R was also higher. Both situations were in line with the results reported by Abe et al. [20]. The QCM-T resonant element reduced the resonant frequency in air by more than 30 kHz after working in the deionized water. The shift in the resonant frequency of the QCM-R was only about 5 kHz because the QCM-R resonant element had a full metal film as a sensing layer on the back of the excitation electrode. This resulted in the metal film acting as a shield such that the electric field could not pass through it into the liquid, which means the frequency variation of the QCM-R resonator was only caused by viscosity and density.

As mentioned above, the electrical properties of the solution were mainly reflected in the permittivity and conductivity, whereas the LFE-QCM was more sensitive to electrical properties than mechanical properties. Measurement of organic solutions of known viscosity, density, and relative permittivity enabled obtaining the frequency shifts of the two resonators. For the experiment, we selected 12 kinds of solutions from dodecane (relative permittivity is 2.012) to pure water (relative permittivity is 80.2), as shown in Table 2.

**Figure 6:**
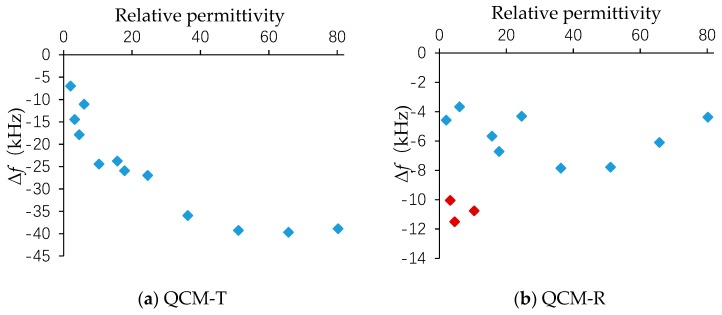
Measured frequency shift difference on the liquid relative permittivity.

Figure 6 shows the relationship between the measured frequency shift Δ*f* and the permittivity. Δ*f* is the fundamental frequency in air minus the resonant frequency in the liquid at room temperature (25 °C). The frequency shift of QCM-T generally shows a certain trend, but several solutions differ from the frequency shift of the adjacent solution. The measured frequency shift of the QCM-R was proportional to the square of the product of the viscosity and density. By carefully comparing the two figures, the deviation point occurred in several solutions where the QCM-R resonator was most sensitive to the viscosity. The points marked with red in Figure 6b are methyloleate, dibutyl sebacate, and 1-octanol. The viscosity of these three solutions was five times more than the viscosity of water, so the frequency shift was higher than that of the other solutions. The frequency difference caused by the simple permittivity could be obtained by subtracting the frequency difference of the QCM-R resonator from the frequency difference of the QCM-T resonator in the same solution.

**Figure 7:**
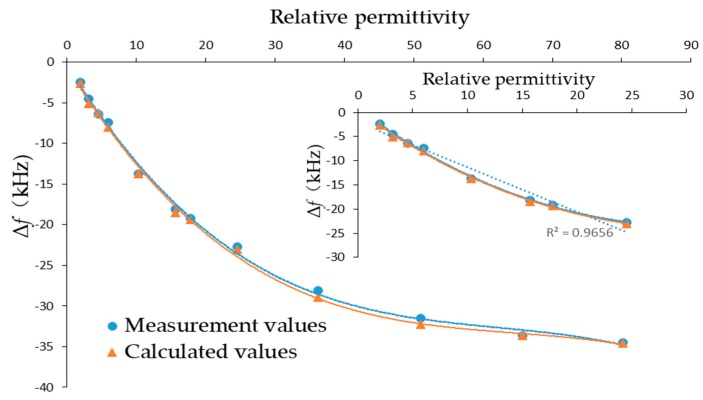
Measurement and calculated frequency shift caused by relative permittivity.

The blue curve shown in Figure 7 is the relationship between the frequency shift caused by the permittivity and the relative permittivity itself, and the fitting degree *R*^2^ was 0.9975 after cubic polynomial fitting for a wide range from 2 to 80. In the low permittivity range, the measurement frequency shift of LFE-QCM had a good linear relationship with the relative permittivity, which also indicated that the QCM was suitable for use as a permittivity sensor in the low permittivity range. The relative permittivity caused a frequency change from 151 to 2157 ppm. Therefore, the above experiments proved that the influence of the viscosity and density could not be neglected when measuring the frequency shift with the lateral field excitation QCM. We also proved that our designed QCM not only had good research value, but also provided a reference for future practical application. According to the Kanazawa formula and the solution viscosity and density, the theoretical frequency shift could be calculated. The yellow curve in Figure 7 represents the measured value minus the theoretical frequency shift calculated using the Kanazawa formula. The coincidence of the two curves also proved that the frequency shift of the QCM-R resonator was only affected by the viscosity and density of the liquid. This result was caused by the smooth gold shield on the QCM-R, which followed well with the Kanazawa formula. Otherwise, a rough gold surface (such as porous gold layer) will induce an increased surface area, which will cause large frequency shift [35].

**Figure 8:**
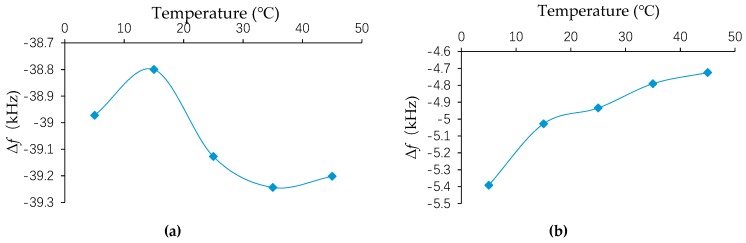

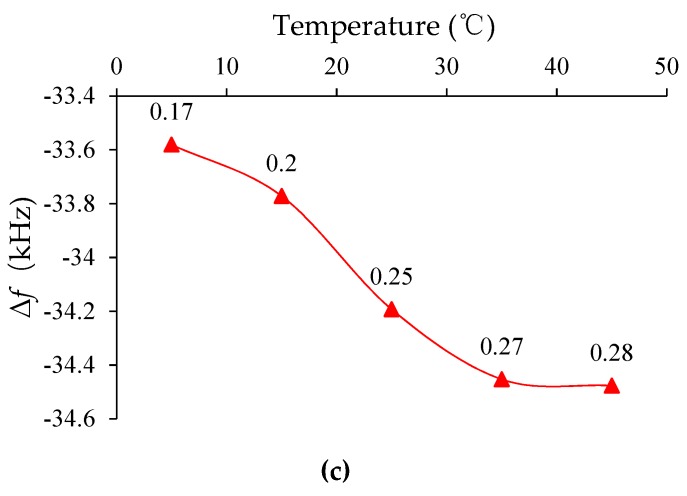
Measured and calculated frequency shift differences caused by temperature. (**a**) Measured frequency shift from QCM-T; (**b**) Measured frequency shift from QCM-R; (**c**) Calculated frequency shift caused by the conductivity.

Similar changes in the frequency shift occurred in the conductivity solution. Especially in practical applications, when the temperature of the liquid or the environment changes, the frequency shift measured by the QCM also changes because the conductivity, liquid viscosity, and density are all affected by temperature. The viscosity and density of the liquid decrease with increasing temperature, and the conductivity increases with increasing temperature within a certain temperature range. Therefore, a 0.01% NaCl solution was selected for this experiment, and the temperature ranged from 5 to 45 °C. The experimental results are shown in Figure 8a,b. The frequency shift of the QCM-R resonant element decreased with the increase in temperature, which agrees with the relationship between viscosity and temperature. The total shift was about 600 Hz. The frequency shift of the QCM-T resonant element caused by temperature did not show an obvious pattern, and the maximum shift difference was about 400 Hz. The total conductivity difference detected by the portable conductivity meter was about 0.1 mS/cm.

However, Figure 8c shows the frequency shift caused by the conductivity alone when the frequency shift between the two resonant elements was subtracted. The frequency shift caused by the conductivity showed a monotonous change with the increase in temperature. Due to the low solubility of NaCl, the content of conductive ions was less. When the temperature rose to 40 °C, the conductivity change was no longer obvious compared with the low temperature, accompanied by a slower frequency shift. The variation pattern also matched the numbers provided in Figure 8c, which were the conductivity values measured using a portable conductivity meter. The conductivity value had a good linear relationship with the frequency shift, which was also consistent with the linear relationship exhibited by the QCM in the low concentration conductivity solution in the literature [16,36]. Therefore, this experiment also proved that the temperature effect had a considerable influence on the measurement of the electrical properties of QCM, and it was not possible to simply neglect the influence of the liquid viscosity and density during the measurement in the liquid.

## 4. Conclusions

The design and fabrication of a dual-channel LFE-QCM was reported in this study. A suitable sensing layer was selected to separate the liquid mechanical properties and electrical properties. The complete QCM chip was designed to be smaller than 13.5 mm × 8 mm and had a fundamental frequency of around 16 MHz. The resonator was characterized by two resonant elements, and its function in air and liquid was studied experimentally. The necessity of the double resonant elements was discussed in terms of the permittivity, conductivity, and temperature of the liquid. Finally, we proved that the influence of liquid viscosity and density should be considered in the application of lateral field excitation QCM and that the structure of the double resonator could eliminate the influence of the environmental temperature on the measurement. Our designed dual-channel LFE-QCM overcame the drawbacks of single channel QCM, which neglects the liquid viscosity and density, and provides a good research basis for the future liquid applications.

## Figures and Tables

**Table 1:** Measured basic properties of dual channel LFE-QCM.

Parameter	In Air		In Deionized Water	
	QCM-T	QCM-R	QCM-T	QCM-R
Resonant Frequency (MHz)	16.494797	16.202182	16.456062	16.196325
Q Value	30671	41743	1267	1127
Conductance (mS)	0.033	10.92	0.178	0.295

**Table 2:** Experimental organic solution characteristics (25 °C).

Solutions	Viscosity (cP)	Density (g/cm^3^)	Relative Permittivity
Dodecane	1.383	0.753	2.012
Methyl oleate	5.5	0.8704	3.21
Dibutyl sebacate	7.96	0.933	4.54
*n*-Propyl acetate	0.59	0.888	6
1-octanol	7.69	0.82143	10.34
Ethyl Acetoacetate	1.5081	1.02126	15.7
Butyl Alcohol	2.95	0.806	17.8
Ethanol	1.103	0.78506	24.55
60% Isopropanol	3.033	0.8786	36.28
40% Isopropanol	2.917	0.9256	51.07
20% Isopropanol	2.059	0.9666	65.72
Pure water	1.002	0.99707	80.2
